# Patterns of Failure After Postoperative Radiotherapy for WHO Grade 2–3 Meningiomas: A Single-Institution Experience

**DOI:** 10.3390/reports9040348

**Published:** 2026-10-13

**Authors:** Mitsutoshi Ooishi, Osamu Togao, Hiroshi Ito, Fumitaka Yoshioka, Tatsuya Abe

**Affiliations:** 1 Department of Radiology, Faculty of Medicine, Saga University, 5-1-1 Nabeshima, Saga 849-8501, Japan; 2 Department of Neurosurgery, Faculty of Medicine, Saga University, 5-1-1 Nabeshima, Saga 849-8501, Japan

**Keywords:** WHO grade 2–3 meningiomas, postoperative radiotherapy, patterns of failure, in-field recurrence

## Abstract

**Background/Objectives**: World Health Organization (WHO) Grade 2–3 meningiomas have high recurrence rates despite postoperative radiotherapy. This study aimed to characterize patterns of failure and clinical outcomes after postoperative external beam radiotherapy. **Methods**: We retrospectively analyzed 10 patients with WHO Grade 2 (n = 6) or Grade 3 (n = 4) meningiomas treated at a single institution between January 2017 and May 2024. Nine patients underwent gross total resection. Radiotherapy was delivered using three-dimensional conformal radiotherapy (n = 6) or intensity-modulated radiotherapy (n = 4), with a median dose of 60 Gy in 30 fractions. Recurrence patterns were assessed relative to the high-dose region. **Results**: The 1- and 3-year overall survival rates were 90% (95% CI, 47–99%) and 79% (95% CI, 38–94%), respectively; corresponding local control rates were 70% (95% CI, 33–89%) and 39% (95% CI, 7–71%), respectively. Recurrence occurred in six patients, predominantly within the high-dose region. Acute toxicities were limited to Grade 1 radiation dermatitis. During follow-up, Grade 3 seizures occurred in three patients, and worsening of pre-existing Grade 2 paresis occurred in four patients. Two seizure events and two cases of worsening paresis were considered more likely related to recurrent or progressive disease, whereas attribution of the remaining neurological events to radiotherapy was uncertain. **Conclusions**: Despite postoperative radiotherapy, local control remained limited in this small, single-institution series, with a predominance of in-field recurrence. This pattern raises the possibility that tumor biological factors, in addition to technical and treatment-related factors, may contribute to treatment failure in WHO Grade 2–3 meningiomas. These findings are exploratory and warrant validation in larger studies incorporating molecular stratification and standardized treatment approaches.

## 1. Introduction

Meningiomas are the most frequently reported histologic type among primary brain and other central nervous system (CNS) tumors, accounting for 42.6% of all such tumors and 57.4% of non-malignant tumors according to the 2025 Central Brain Tumor Registry of the United States (CBTRUS) Statistical Report [1]. Meningiomas had the highest incidence of any primary CNS tumor type (approximately 10 per 100,000 population per year) [1]. These tumors exhibit a female predominance and are most often diagnosed during the fourth to seventh decades of life, with a peak in incidence around age 65 [2]. Most meningiomas are classified as World Health Organization (WHO) Grade 1 (benign), accounting for roughly 95% of cases, whereas Grade 2 and Grade 3 tumors comprise approximately 4% and 1%, respectively [3]. Consistent with these trends, a nationwide cohort from the Brain Tumor Registry of Japan (BTRJ)—including 4081 patients who underwent initial resection for intracranial meningiomas between 2001 and 2008—reported WHO Grade 1, 2, and 3 tumors in 92.4%, 6.3%, and 1.3% of cases, respectively [4]. Meningiomas are currently classified according to the 2021 World Health Organization (WHO) Classification of Tumors of the Central Nervous System, which integrates both histopathological and molecular criteria. Grade 1 tumors exhibit benign biological behavior; Grade 2 tumors are defined by increased mitotic activity, brain invasion, or aggressive histologic variants such as chordoid or clear cell subtypes; and Grade 3 tumors demonstrate overtly malignant morphology or harbor high-risk molecular alterations. Notably, TERT promoter mutations and CDKN2A/B homozygous deletions are recognized as defining molecular features of CNS WHO Grade 3 meningiomas [5]. Recent cIMPACT-NOW recommendations have further refined meningioma grading by providing guidance on standardized morphological assessment and the interpretation of molecular risk parameters, underscoring the growing role of molecular features in risk stratification [6]. Treatment strategies vary according to tumor size, symptomatology, anatomical constraints, and biological grade. Observation may be appropriate for small or asymptomatic meningiomas; however, intervention becomes necessary when symptoms are present or anticipated, or when patients exhibit tumor- or treatment-related risk factors, such as proximity to the optic nerve [7]. Surgical resection remains the cornerstone of management. In clinical practice, Grade 1 tumors are commonly treated with surgery alone or stereotactic radiosurgery when feasible. For Grade 2 meningiomas, adjuvant radiotherapy is considered—even after gross total resection—due to the substantial risk of recurrence. For Grade 3 meningiomas, postoperative radiotherapy is generally indicated regardless of the extent of resection because of their highly aggressive biological behavior. Despite these established treatment paradigms, high-grade meningiomas remain challenging to manage, with recurrence rates historically reported to be approximately 30–50% for Grade 2 tumors and 50–90% for Grade 3 tumors in earlier retrospective series [8]. Multiple predictors of recurrence have been identified, including pathological features such as the Ki-67 proliferation index, brain invasion, and bone invasion, as well as clinical factors such as extent of resection [9,10]. More recently, machine learning and radiomics-based approaches have been investigated as tools for predicting meningioma recurrence, reflecting increasing interest in multidimensional risk stratification [11]. While several studies have reported clinical and pathological predictors of recurrence, limited data are available regarding how these factors translate into spatial patterns of failure after postoperative radiotherapy. Therefore, we retrospectively evaluated patients with WHO Grade 2 and Grade 3 meningiomas treated with postoperative external beam radiotherapy at our institution, with a specific focus on spatial failure patterns. 

## 2. Materials and Methods

### 2.1. Patient Characteristics

This retrospective study was approved by the Institutional Review Board of Saga University Hospital (Approval No. 2024-10-R-01). Informed consent was not required in accordance with institutional policies for retrospective studies. We analyzed 10 patients with World Health Organization (WHO) Grade 2 or Grade 3 meningiomas who received postoperative external beam radiotherapy at Saga University Hospital between January 2017 and May 2024. All pathological diagnoses were reviewed in accordance with the 2021 WHO classification. These 10 patients represented all consecutive patients who met the predefined eligibility criteria during the study period, and no additional exclusion criteria were applied. Molecular analysis for TERT promoter mutation and CDKN2A/B homozygous deletion was performed in all 10 patients. Among the 10 patients, six had Grade 2 tumors and four had Grade 3 tumors. The median age at radiotherapy was 75 years (range, 38–85 years), and the cohort consisted of six males and four females. Performance status (PS), assessed using the Eastern Cooperative Oncology Group (ECOG) scale, was 0 in four patients, 1 in three patients, and 2, 3, and 4 in one patient each. The patient with PS 3 had a history of brainstem infarction and presented with incomplete paralysis of the left upper and lower extremities prior to the initiation of treatment, whereas the patient with PS 4 had profound intellectual disability. Tumor locations included the parasagittal region in four patients, the frontal lobe in two patients, and the skull base, convexity, suprasellar region, and lateral ventricle in one patient each. Six patients underwent surgery for primary disease, whereas four underwent surgery for recurrent disease prior to postoperative radiotherapy. Preoperative endovascular embolization was performed in four cases. The extent of resection was determined based on operative records and postoperative imaging findings and was further classified according to the Simpson grading system. Gross total resection was defined as the absence of visible residual intracranial tumor and corresponded to Simpson grades I–III, whereas Simpson grade IV was classified as subtotal resection. In cases involving the venous sinus, dura, or adjacent bone, classification as gross total resection did not necessarily indicate complete removal of all involved dural, osseous, or sinus tissues. When the tumor involved a major dural venous sinus, resection of the involved sinus portion was attempted when considered surgically safe, taking into account the adequacy of collateral venous drainage; otherwise, the involved sinus was preserved. In cases of bone invasion, involved bone was resected as extensively as safely feasible. The median tumor volume, measured on contrast-enhanced MRI immediately prior to the final surgical resection performed before radiotherapy, was 50.6 cm^3^ (range, 28.1–103.7 cm^3^).

### 2.2. Radiotherapy Planning

Patients were immobilized in the supine position using a custom-made thermoplastic mask. Simulation CT was performed with a slice thickness of 1–2 mm. Radiotherapy was delivered using 6-MV photon beams. Treatment planning was performed with Pinnacle^3^ (Philips Radiation Oncology Systems, Fitchburg, WI, USA) until July 2021 and with RayStation (RaySearch Laboratories, Stockholm, Sweden) thereafter. Radiotherapy was administered using clinically available linear accelerators, including the Oncor Impression (Siemens Healthineers, Erlangen, Germany), the Versa HD system (Elekta AB, Stockholm, Sweden), and the Radixact X7 TomoTherapy system (Accuray Incorporated, Sunnyvale, CA, USA). All patients received conventionally fractionated radiotherapy, with a median total dose of 60 Gy in 30 fractions (range, 45–60 Gy in 25–30 fractions). One 38-year-old male patient with a remote history of prophylactic whole-brain irradiation for acute myeloid leukemia received 45 Gy in 25 fractions to limit the cumulative dose from prior cranial irradiation. His prior cranial irradiation consisted of 22.5 Gy administered more than three decades earlier as part of post-chemotherapy prophylaxis. Radiotherapy techniques included three-dimensional conformal radiotherapy (3DCRT) in six patients and intensity-modulated radiotherapy (IMRT) in four patients. Among the patients treated with IMRT, one patient was treated with step-and-shoot fixed-field IMRT, one with volumetric modulated arc therapy (VMAT), and two with TomoTherapy. Nine of the 10 patients underwent MRI prior to radiotherapy. Contrast-enhanced MRI was performed in six of these nine patients. In the remaining three patients, non-contrast MRI included T1-weighted, T2-weighted, fluid-attenuated inversion recovery (FLAIR), diffusion-weighted imaging (DWI), and high-resolution T2-weighted sampling perfection with application-optimized contrasts using different flip-angle evolutions (SPACE) sequences with a slice thickness of 0.4 mm. These sequences, particularly the high-resolution T2-weighted SPACE images, were used as reference images for treatment planning and anatomical assessment. The patient without pre-radiotherapy MRI had undergone subtotal resection and had a preoperative contrast-enhanced MRI as well as a head CT obtained before radiotherapy planning. In this patient, the preoperative contrast-enhanced MRI, pre-radiotherapy head CT, and radiotherapy planning CT were reviewed together for target delineation. When contrast-enhanced pre-radiotherapy MRI was unavailable, target delineation was performed by integrating all available imaging studies with operative findings and pathological information to identify the postoperative tumor bed and anatomical regions at risk for microscopic extension. Target volumes were defined according to the extent of surgical resection. The gross tumor volume (GTV) was defined as all visible residual disease and was delineated in the single case of subtotal resection (STR) included in this cohort. The clinical target volume (CTV) included anatomical regions considered at risk for microscopic tumor spread, comprising the preoperative dural attachments, suspected dural tail extension, areas of invasion into the brain parenchyma or bone, and tumor-induced hyperostosis identified on pre- and postoperative MRI, when available. In addition, informed by pathological findings and detailed consultation with the operating neurosurgeon, the extent of tumor infiltration—including dural involvement and parenchymal invasion—was thoroughly assessed. In principle, the CTV was generated by applying a 5–10 mm margin to all relevant target components, including the GTV (in the STR case) and the aforementioned anatomical regions at risk. In cases of gross total resection (GTR), no GTV was defined, and the CTV was delineated based on the postoperative resection cavity and surrounding regions at risk. The planning target volume (PTV) was generated by expanding the CTV by an additional uniform 5-mm margin. These target delineation principles, including the use of a 5–10 mm CTV margin, remained generally consistent throughout the study period despite changes in treatment planning systems and radiotherapy delivery techniques.

### 2.3. Endpoints and Statistical Analysis

Follow-up was calculated from the date of completion of radiotherapy. Overall survival and local control rates at 1 and 3 years were estimated. Local failure was defined on serial follow-up imaging as the appearance of a new lesion or progressive enlargement within or adjacent to the postoperative resection cavity or treated tumor bed. After gross total resection, local failure required the appearance of a new lesion within or adjacent to the resection cavity with subsequent enlargement on follow-up imaging. In the single case of subtotal resection, local failure was defined as progressive enlargement of the residual tumor compared with the post-radiotherapy baseline imaging. No predefined quantitative or volumetric threshold, such as RECIST or RANO criteria, was applied. Scheduled follow-up imaging was performed using MRI. Follow-up MRI protocols generally included T1-weighted, T2-weighted, FLAIR, diffusion-weighted imaging (DWI), and high-resolution T2-weighted SPACE sequences. Contrast-enhanced fat-suppressed T1-weighted imaging and contrast-enhanced fat-suppressed T1-weighted volumetric interpolated breath-hold examination (VIBE) sequences were also obtained when clinically appropriate and available. CT was additionally obtained when clinically indicated because of acute neurologic symptoms, such as dizziness, headache, suspected seizure, or altered consciousness. Overall survival and local control were estimated using the Kaplan–Meier method with SPSS software (version 28; IBM SPSS Statistics for Windows, IBM Corp., Armonk, NY, USA). For the LC analysis, local failure was considered the event. Death without prior local failure would have been treated as a censoring event; however, no such deaths occurred in this cohort. Therefore, a competing-risks analysis was not performed. Two-sided 95% confidence intervals (CIs) were calculated for the Kaplan–Meier estimates of local control and overall survival. Given the small sample size and limited number of events, no formal subgroup survival comparisons or log-rank tests according to WHO grade or Ki-67 category were performed; these factors were evaluated descriptively. For the same reason, no formal sensitivity analysis based on alternative follow-up restrictions or subgroup exclusions was performed, as such analyses would have resulted in very small subsets and unstable estimates. Adverse events were recorded according to CTCAE version 5.0 [12]. Events occurring after radiotherapy were documented regardless of causality; however, for neurological events, attribution to radiotherapy was assessed individually based on baseline neurological function, seizure history, antiepileptic medication use, tumor recurrence or progression, and follow-up imaging findings. Imaging findings potentially related to treatment effects, including changes in cerebral edema or T2/FLAIR signal abnormalities, white matter changes, and radiation necrosis, were also reviewed. When alternative causes were present or a definitive cause could not be established, the relationship between the neurological event and radiotherapy was considered uncertain. Patterns of failure were assessed by reviewing follow-up imaging at the time of recurrence in conjunction with the original radiotherapy treatment plans. For the qualitative classification of recurrence patterns, the spatial relationship between recurrent lesions and the radiotherapy dose distribution was evaluated visually using the planning system. Recurrences were classified relative to the 90% isodose line of the prescribed dose. The 90% isodose line was selected in accordance with prior pattern-of-failure analysis in postoperative meningioma radiotherapy [13]. In-field recurrence was defined as recurrence occurring predominantly within the 90% isodose of the prescribed dose; marginal recurrence was defined as recurrence arising adjacent to but predominantly outside the 90% isodose line; and out-of-field recurrence was defined as recurrence developing at anatomically distant sites beyond the irradiated region. To provide an objective quantitative assessment of these spatial relationships, recurrent tumor volumes were delineated on the follow-up MRI sequence that most clearly depicted each recurrent lesion. Depending on lesion conspicuity and available imaging, contrast-enhanced fat-suppressed T1-weighted VIBE, contrast-enhanced fat-suppressed T1-weighted imaging, or high-resolution T2-weighted SPACE imaging was used for delineation. The follow-up MRI was rigidly co-registered with the original radiotherapy planning CT, and the spatial relationship between each recurrent tumor volume and the original dose distribution was quantitatively evaluated. For each recurrent lesion, the recurrent tumor volume, V90, defined as the percentage of the recurrent tumor volume receiving at least 90% of the prescribed dose, and D95, defined as the dose received by at least 95% of the recurrent tumor volume, were calculated. In one patient with both marginal and out-of-field recurrences, the two recurrent lesions were delineated and analyzed separately. Recurrence classification was not performed independently; rather, the final classification was determined by consensus review of follow-up imaging and treatment plans by the treating radiation oncologists and radiologists. Any differences in interpretation were discussed jointly until a consensus classification was reached, and no unresolved disagreement remained regarding the final classification.

## 3. Results

### 3.1. Patient Characteristics and Treatment Outcomes

**Figure 1:**
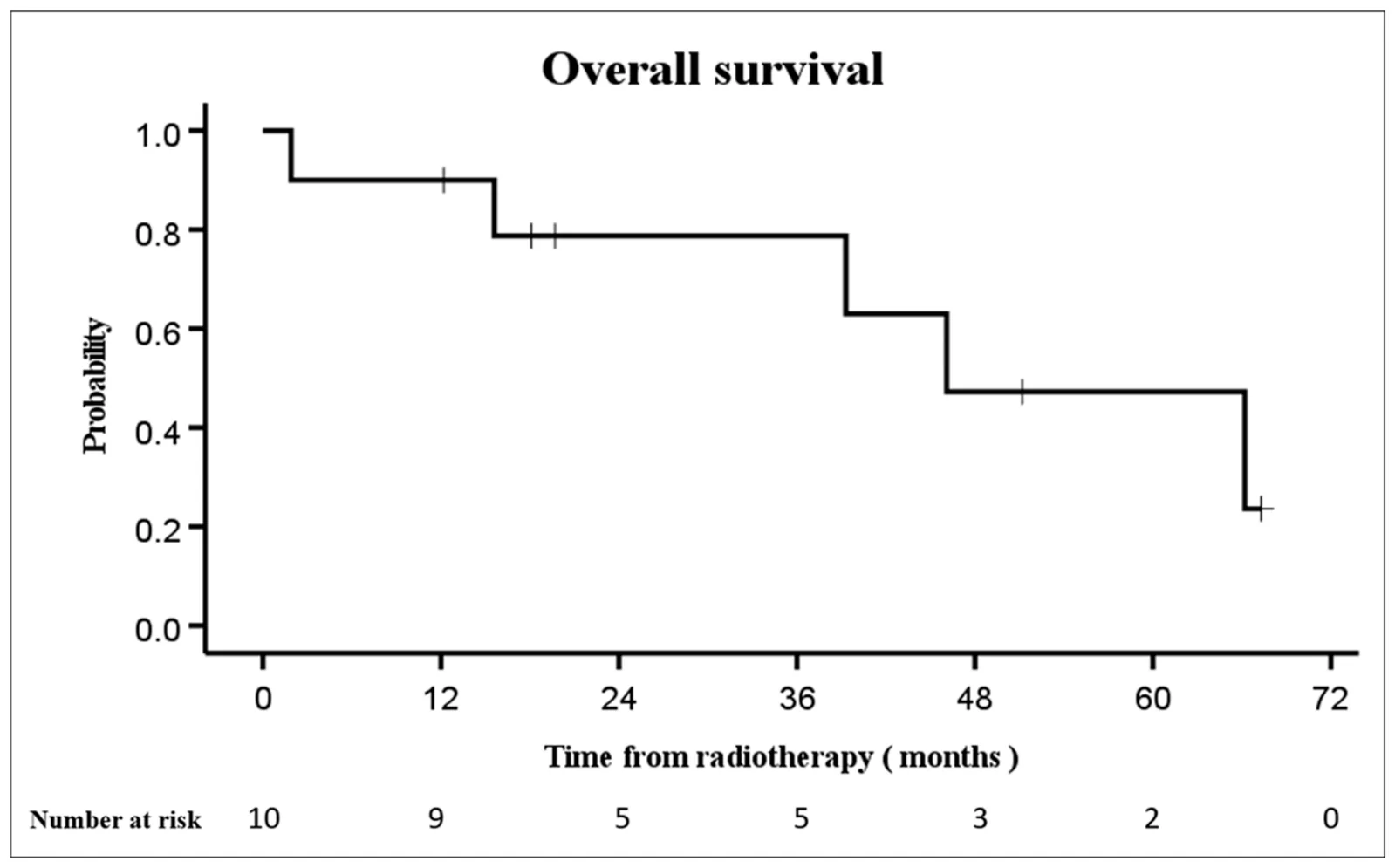
Overall survival after postoperative conventionally fractionated radiotherapy for World Health Organization (WHO) Grade 2 and 3 meningiomas. Numbers at risk at selected time points are shown below the Kaplan–Meier curve. The short vertical tick marks indicate censored observations.

**Figure 2:**
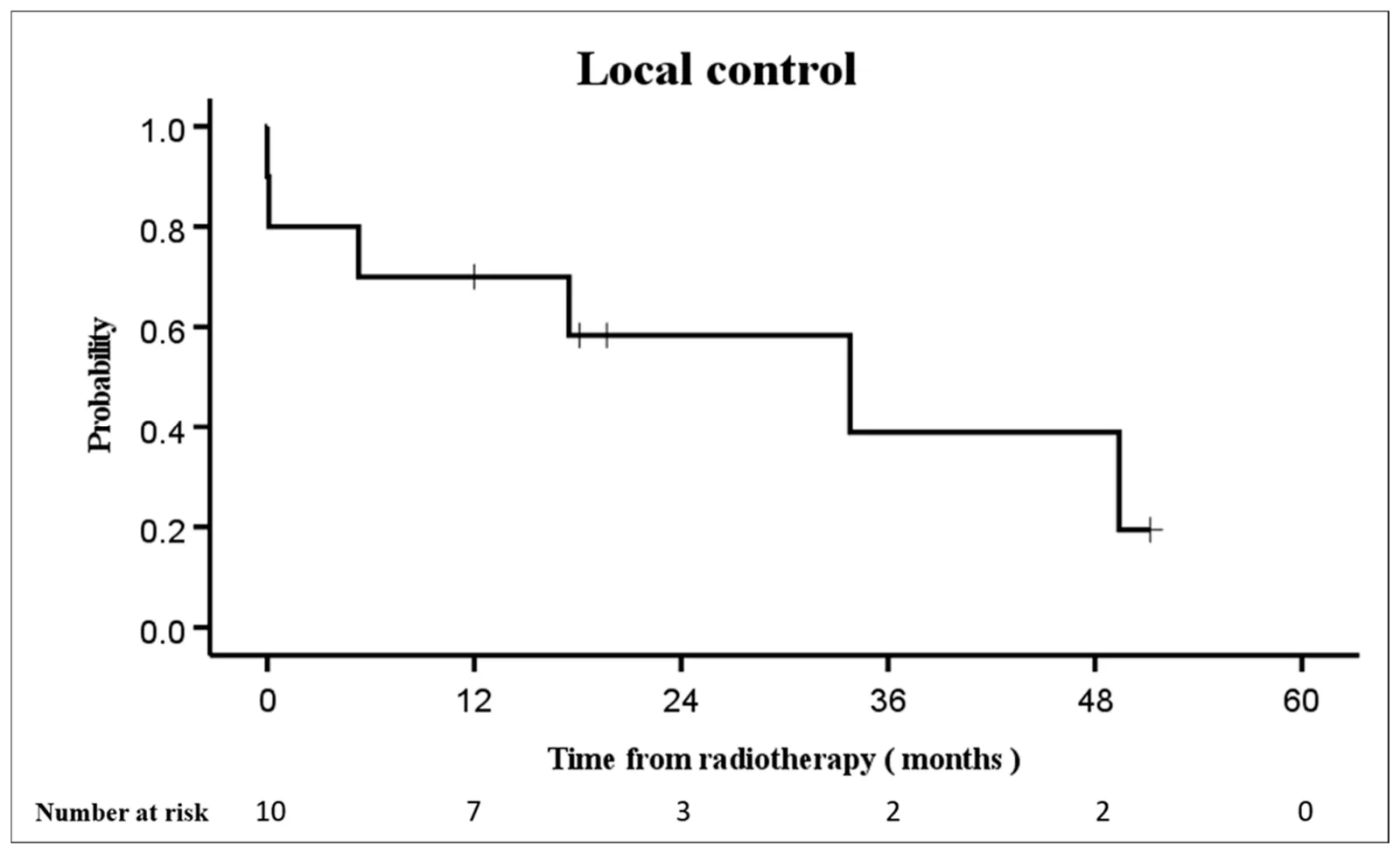
Local control after postoperative conventionally fractionated radiotherapy for WHO Grade 2 and 3 meningiomas. Numbers at risk at selected time points are shown below the Kaplan–Meier curve. The short vertical tick marks indicate censored observations.

Gross total resection was achieved in nine patients, whereas one patient underwent subtotal resection. According to the Simpson grading system, four patients had grade I resection, two had grade II resection, three had grade III resection, and one had grade IV resection. TERT promoter mutation and CDKN2A/B homozygous deletion were assessed in all 10 patients, and neither alteration was detected in any patient. The median follow-up period was 29.5 months (range, 1.9–67.3 months). The patient with the shortest follow-up period of 1.9 months died of the primary disease shortly after radiotherapy and was not lost to follow-up. The 1-year overall survival rate was 90% (95% CI, 47–99%), decreasing to 79% (95% CI, 38–94%) at 3 years (Figure 1). The 1-year local control rate was 70% (95% CI, 33–89%), declining to 39% (95% CI, 7–71%) at 3 years (Figure 2).

Recurrence occurred in six of the 10 patients. Four presented with either WHO Grade 3 histology or recurrent disease at initial presentation. For descriptive purposes, recurrence outcomes were also examined according to the Ki-67 proliferation index. Recurrence occurred in 2 of 3 patients with a Ki-67 index < 10%, 1 of 2 patients with a Ki-67 index of 10–29%, and 3 of 5 patients with a Ki-67 index ≥ 30%. The median Ki-67 index was 22.5% (range, 5–69%) among patients with recurrence and 22.5% (range, 5–30%) among those without recurrence. No formal statistical comparison was performed because of the small number of patients in each subgroup. The median time from radiotherapy to recurrence was 11.4 months (range, 0–49.4 months). The most common pattern of recurrence was local recurrence (Table 1).

Using the 90% isodose line as the reference, five of the six patients with recurrence had in-field failure. The remaining patient had one marginal and one out-of-field recurrent lesion. Quantitative dosimetric analysis showed that V90 ranged from 87.5% to 100% among the five in-field recurrent lesions. In contrast, the marginal recurrent lesion had a V90 of 1.5%, whereas the out-of-field recurrent lesion showed no overlap with the 90% prescription isodose volume (V90 = 0%). Detailed volumetric and dosimetric characteristics of the recurrent lesions are summarized in Table 2.

**Figure 3:**
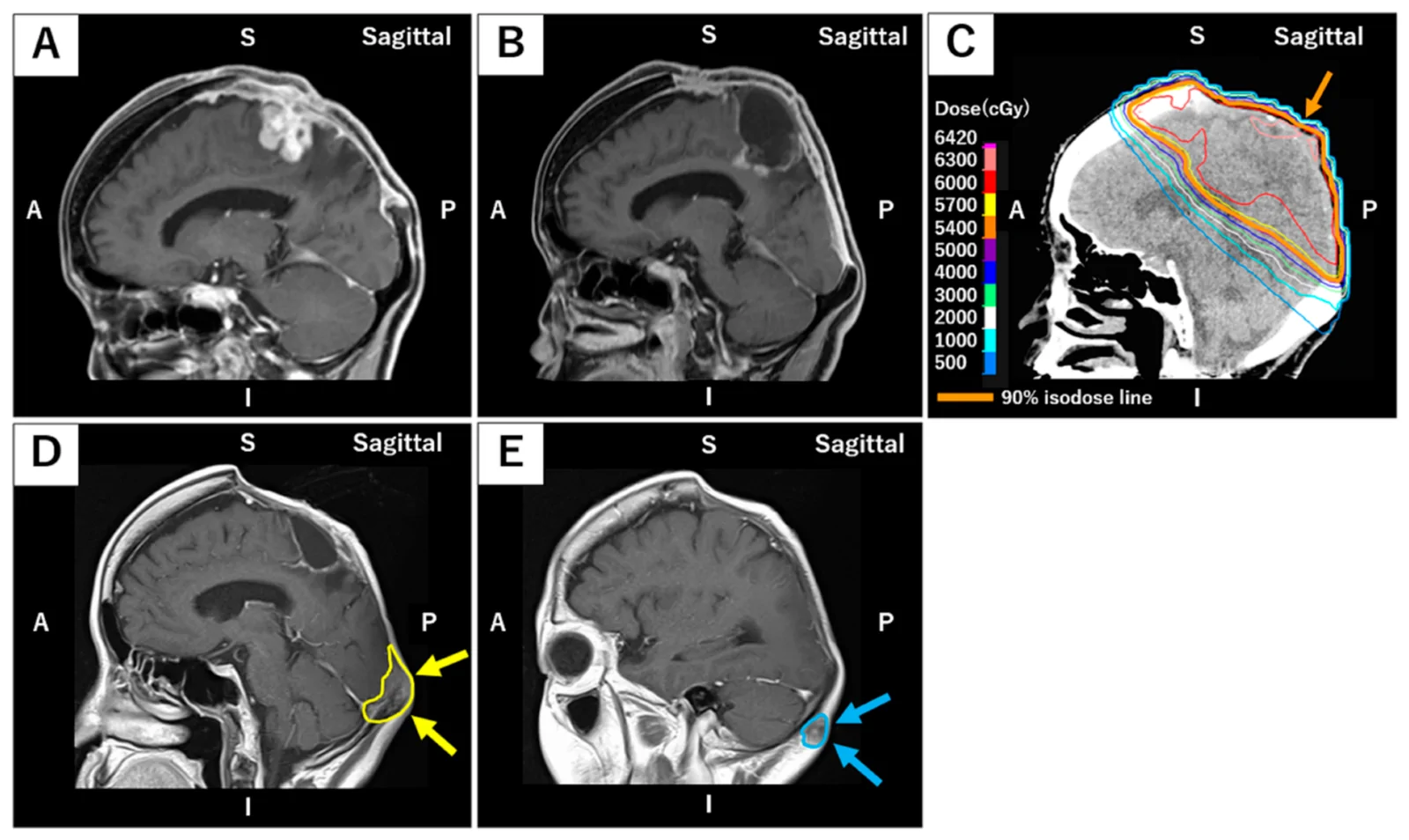
Marginal and out-of-field recurrence in a 66-year-old female patient with recurrent WHO Grade 3 parasagittal meningioma. The patient had undergone two prior surgical resections, with the most recent achieving gross total resection. Histopathological examination demonstrated brain and bone invasion, with a Ki-67 index of 40%. (**A**) Pre-radiotherapy contrast-enhanced T1-weighted sagittal MRI showing a well-defined enhancing recurrent tumor in the parietal region, along with an additional lesion involving the occipital bone. (**B**) Postoperative contrast-enhanced T1-weighted MRI obtained immediately prior to radiotherapy, demonstrating no evidence of residual disease. (**C**) Dose distribution of 3DCRT delivering 60 Gy in 30 fractions. The 20 Gy isodose line is shown in white, the 40 Gy isodose line in blue, and the 60 Gy isodose line in red. The orange line indicates the 90% prescription isodose (54 Gy), highlighted by the orange arrow. (**D**,**E**) Contrast-enhanced T1-weighted MRI obtained approximately 5.5 months after radiotherapy, demonstrating a marginal recurrence in the occipital bone (yellow outline and arrows) and an out-of-field recurrence in the subcutaneous tissue of the posterior neck (light blue outline and arrows). Quantitative volumetric analysis showed that 1.5% of the marginal recurrent tumor volume overlapped the 90% prescription isodose volume, indicating that the lesion was predominantly located outside the high-dose region. The out-of-field recurrent lesion showed no overlap with the 90% prescription isodose volume. Both lesions were subsequently treated with salvage surgery. Orientation labels: A, anterior; P, posterior; S, superior; I, inferior.

Among these six patients, salvage treatment was administered in four cases. Salvage surgery was performed at our institution in three patients, whereas Gamma Knife radiosurgery was performed at another institution in one patient. The remaining two patients received best supportive care because of poor performance status. Of the four patients who underwent salvage treatment, three ultimately died of the primary disease, whereas one patient remains alive. When stratified by WHO grade, recurrence occurred in three of four patients with Grade 3 meningiomas and in three of six patients with Grade 2 meningiomas. In-field recurrence was the predominant pattern, observed in two of three recurrent Grade 3 cases and in all three recurrent Grade 2 cases. One patient experienced both marginal recurrence and out-of-field recurrence, with the latter involving the right posterior cervical subcutaneous region. This patient was a 66-year-old woman with recurrent WHO Grade 3 parasagittal meningioma who had undergone two prior surgeries before radiotherapy, with the most recent operation achieving gross total resection. Histopathological examination revealed brain and bone invasion with a Ki-67 index of 40%. She was treated with three-dimensional conformal radiotherapy (3DCRT) to a total dose of 60 Gy in 30 fractions and developed recurrence approximately 5.5 months after radiotherapy, consisting of a marginal recurrence in the occipital bone and an out-of-field recurrence in the posterior cervical subcutaneous tissue (Figure 3). Both recurrent lesions were treated with salvage surgery; however, approximately 5 months after salvage surgery, cervical lymph node metastasis developed. No further aggressive treatment was pursued, and best supportive care was initiated.

**Figure 4:**
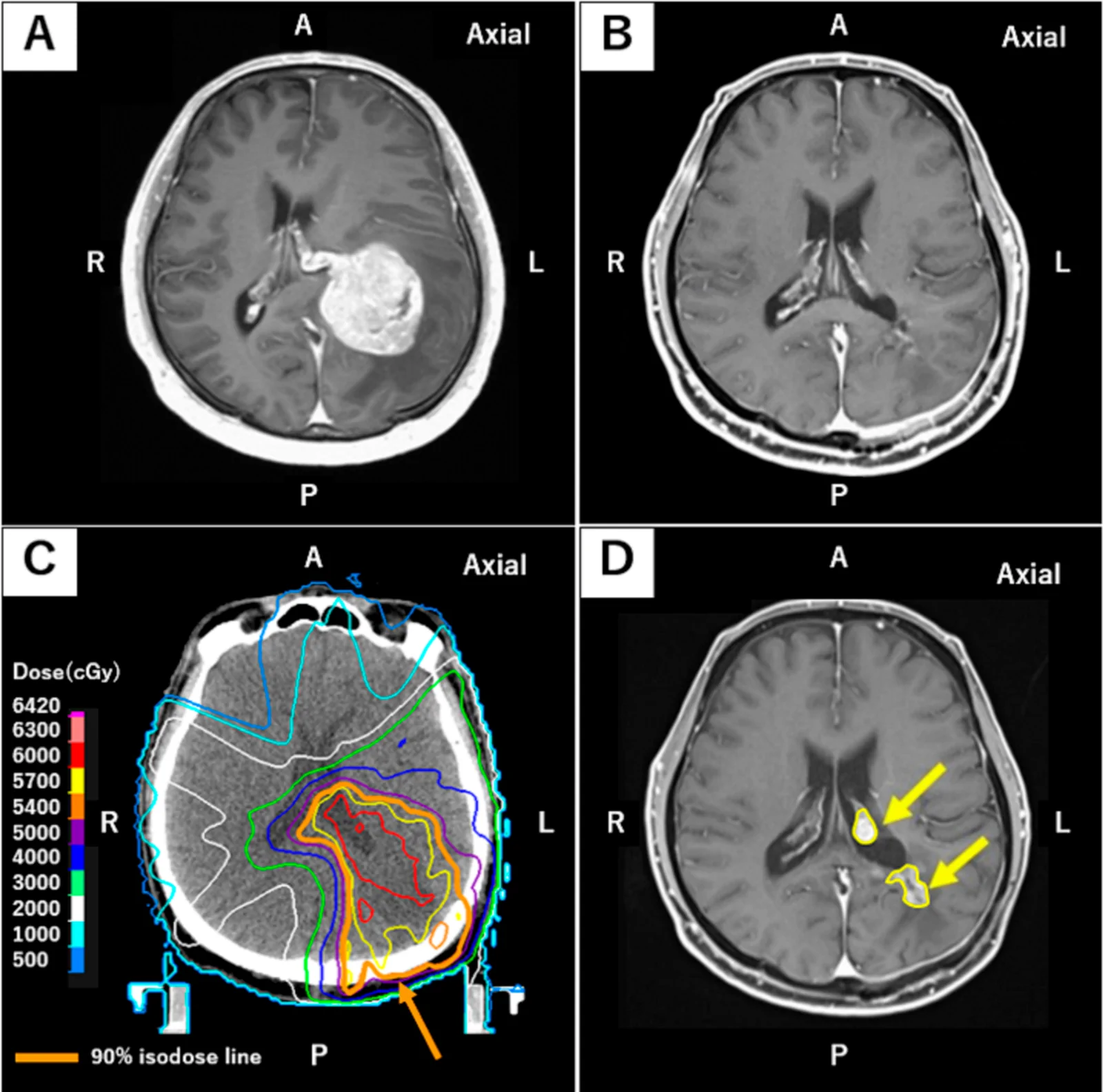
In-field recurrence in a 44-year-old male patient diagnosed with WHO Grade 2 meningioma. The tumor was completely resected (gross total resection) and exhibited brain invasion, with a Ki-67 index of 31%. (**A**) Preoperative contrast-enhanced T1-weighted MRI showing a well-demarcated tumor in the left lateral ventricular trigone, measuring 56 mm × 46 mm × 37 mm. (**B**) Postoperative contrast-enhanced T1-weighted MRI obtained prior to radiotherapy demonstrating complete resection with no residual disease. (**C**) Dose distribution for intensity-modulated radiotherapy (IMRT), planned to deliver a total dose of 60 Gy in 30 fractions. The 20 Gy isodose line is displayed in white, the 40 Gy isodose line in blue, and the 60 Gy isodose line in red. The orange line indicates the 90% prescription isodose (54 Gy), highlighted by the orange arrow. (**D**) Axial contrast-enhanced T1-weighted MRI acquired approximately 6 months after radiotherapy, showing recurrent tumor in the left lateral ventricular trigone and involvement of the choroid plexus at the level of the ventricular body (yellow outline and arrows). The recurrence was located entirely within the 90% isodose volume. Quantitative volumetric analysis showed that 100% of the recurrent tumor volume overlapped the 90% prescription isodose volume. The patient declined reoperation and was treated with salvage Gamma Knife radiosurgery. Orientation labels: A, anterior; P, posterior; R, right; L, left.

Figure 4 presents a representative case of in-field recurrence. The patient was a 44-year-old man diagnosed with a WHO Grade 2 meningioma. Gross total resection was achieved, and histopathological examination demonstrated brain invasion with a Ki-67 index of 31%. Despite postoperative IMRT to a total dose of 60 Gy in 30 fractions, follow-up imaging approximately 6 months after radiotherapy revealed tumor recurrence in the left trigone of the lateral ventricle, involving the choroid plexus at the level of the ventricular body. Salvage surgery was declined, and Gamma Knife radiosurgery was subsequently performed.

### 3.2. Toxicities

In all patients, radiotherapy was completed without interruption. Acute toxicities were limited to Grade 1 radiation dermatitis, which was observed in seven patients. A documented history of seizures before radiotherapy was present in two of the 10 patients, and pre-existing Grade 2 paresis was present in four patients. During follow-up after radiotherapy, Grade 3 seizures occurred in three patients. In two patients, the seizure events were considered more likely related to recurrent or progressive disease than to radiotherapy. In the remaining patient, the causal relationship with radiotherapy remained uncertain. The patient in whom the relationship with radiotherapy remained uncertain was an 80-year-old woman with a pre-existing history of symptomatic epilepsy at the time of initial presentation to our hospital, likely attributable to the primary disease. Following radiotherapy, she was maintained on levetiracetam at a dose of 1000 mg/day. Approximately 2 years after radiotherapy, she experienced a seizure episode, which was managed medically by increasing the levetiracetam dose to 1500 mg/day. A similar transient seizure episode occurred approximately 8 months later, after which the levetiracetam dose was further increased to 2000 mg/day. Since this dose adjustment, no further seizure events have been observed. Grade 1 cognitive disturbance was subsequently documented. Follow-up MRI showed no clear increase in local cerebral edema, no focal progression of T2/FLAIR abnormalities, and no radiation necrosis. Although white matter changes became more apparent during follow-up, their relationship to radiotherapy was uncertain. Of the two patients whose seizures were considered more likely disease-related, one had no history of seizures but had been receiving levetiracetam at a dose of 1000 mg/day since surgery. The medication was discontinued approximately 8 months after radiotherapy because of a stable clinical course. Approximately 6 months after discontinuation, the patient experienced a generalized seizure of uncertain semiology during sleep, and levetiracetam at 1000 mg/day was subsequently reintroduced. MRI around the time of the seizure showed findings that raised the possibility of post-treatment changes, including suspected necrotic or hemorrhagic change, although tumor recurrence could not be excluded. There was no worsening of cerebral edema. However, the enhancing lesion subsequently enlarged and, in retrospect, was considered more consistent with tumor recurrence. Although discontinuation of antiepileptic medication may also have contributed to seizure occurrence, the seizure was considered more likely related to tumor recurrence or progression than to radiotherapy. The second patient whose seizure was considered more likely disease-related developed a Grade 3 seizure in the setting of progressive recurrent tumor. MRI demonstrated enlargement of the recurrent lesion without worsening cerebral edema or radiation necrosis; therefore, this event was also considered more likely related to tumor progression than to radiotherapy.

Pre-existing Grade 2 paresis was present in four patients. In two patients, subsequent neurological worsening occurred in association with tumor recurrence or progression and was considered more likely disease-related. In the remaining two patients, worsening of pre-existing Grade 2 paresis occurred without clear evidence of tumor progression. One patient was an 85-year-old woman with a WHO Grade 3 meningioma involving the left aspect of the superior sagittal sinus who underwent postoperative IMRT to a total dose of 60 Gy in 30 fractions. She had Grade 2 right upper- and lower-extremity paresis before radiotherapy. Beginning approximately 1 year after radiotherapy, the paresis gradually worsened, and Grade 1 cognitive disturbance also developed. MRI showed no increase in cerebral edema, no progression of T2/FLAIR abnormalities, no radiation necrosis, and no tumor recurrence; therefore, the causal relationship with radiotherapy remained uncertain. The paresis was managed conservatively. The other patient was a 76-year-old man who received postoperative IMRT (60 Gy in 30 fractions) for a recurrent WHO Grade 2 meningioma involving the left aspect of the superior sagittal sinus. This patient had already demonstrated Grade 2 right lower-extremity weakness prior to radiotherapy. Although the weakness gradually worsened during follow-up, MRI showed neither increased cerebral edema nor radiation necrosis, and deconditioning was also considered a possible contributing factor. Accordingly, the worsening weakness could not be unequivocally attributed to radiotherapy. Overall, although neurological events were observed during follow-up, none could be unequivocally attributed to radiotherapy.

## 4. Discussion

In this single-institution series, postoperative radiotherapy for WHO Grade 2–3 meningiomas resulted in limited local control, with a clear predominance of in-field recurrence. Comparison with prospective cooperative group trials and historical series suggests that these outcomes may partly reflect the enrichment of high-risk biological features, although technical and treatment-related factors cannot be excluded. This study should be interpreted as a hypothesis-generating, descriptive analysis reflecting real-world clinical practice in a high-risk population, rather than as a definitive comparative or statistically powered study. The management of WHO Grade 2 and 3 meningiomas remains challenging, with persistently high recurrence rates despite advances in surgical and radiotherapy techniques [8]. Surgical resection remains the cornerstone of initial management, with the primary goal of achieving maximal safe tumor removal. Although postoperative radiotherapy is employed to improve local control [14], recurrence remains a substantial clinical challenge, particularly in biologically aggressive high-grade meningiomas. Importantly, the indication for postoperative radiotherapy differs substantially according to tumor grade. For WHO Grade 2 meningiomas, the role of adjuvant radiotherapy remains nuanced and highly dependent on clinicopathologic risk factors, particularly the extent of resection. Several retrospective studies have reported that postoperative radiotherapy is not associated with a significant improvement in overall survival among patients who undergo gross total resection [15,16], underscoring the ongoing controversy regarding routine adjuvant treatment in this subgroup. In contrast, for WHO Grade 3 meningiomas, adjuvant radiotherapy is generally regarded as standard of care, reflecting their aggressive clinical behavior and consistently high rates of early recurrence.

Prospective cooperative group trials provide the strongest contemporary benchmarks for postoperative radiotherapy in high-grade meningiomas. In NRG Oncology RTOG 0539, intermediate-risk patients—defined as those with WHO Grade 2 meningiomas after gross total resection or recurrent or subtotally resected WHO Grade 1 tumors—achieved excellent outcomes with postoperative radiotherapy to 54 Gy, with a 3-year progression-free survival (PFS) of 93.8% and a local failure rate of only 4.1% [17]. In contrast, high-risk patients, including those with WHO Grade 3 meningiomas or subtotally resected or recurrent WHO Grade 2 tumors, demonstrated more modest outcomes despite dose escalation to 60 Gy delivered with intensity-modulated radiotherapy, with a 3-year PFS of 59.2% and local control of 68.9% [18]. Similarly, the EORTC 22042-26042 trial, which focused on a more selected population of patients with newly diagnosed WHO Grade 2 meningiomas following complete resection, reported a favorable 3-year PFS of 88.7% with adjuvant radiotherapy to 60 Gy [19]. Taken together, these prospective data suggest that while postoperative radiotherapy provides meaningful disease control in selected intermediate-risk settings, outcomes remain suboptimal in high-risk populations.

Several retrospective studies further contextualize these findings and reinforce the role of postoperative radiotherapy in high-grade meningiomas. Detti et al. analyzed 68 patients treated with postoperative radiotherapy for atypical and malignant meningiomas and reported favorable long-term outcomes, with 5-year actuarial overall survival (OS) and disease-free survival (DFS) rates of 74.1% and 76.5%, respectively. In their series, OS was independently associated with radiation dose ≥52 Gy, whereas DFS was primarily influenced by tumor size and histological grade rather than dose [20]. In a 25-year single-institution analysis, Kent et al. also showed significantly improved progression-free survival with surgery plus postoperative radiotherapy compared with surgery alone (median 5.9 vs. 2.1 years), as well as superior 5-year OS rates (71.2% vs. 66.3%) [21]. As summarized in Table 3, comparison with historical series demonstrates that the markedly lower 3-year local control rate of 39% observed in our cohort may partly reflect an enrichment for adverse prognostic features, including a substantial proportion of WHO Grade 3 tumors, frequent cases with elevated Ki-67 indices, a high incidence of brain invasion, and several patients treated for recurrent disease.

Collectively, these factors may have contributed to the relatively unfavorable local control observed in our cohort. Taken together, these findings provide a rationale for further investigation of individualized treatment approaches, including treatment intensification and multimodal strategies, in selected high-risk patients.

Across multiple retrospective studies, atypical and anaplastic meningiomas have been reported to carry a substantial risk of local relapse, with recurrence rates broadly estimated at 29–52% in WHO Grade 2 tumors and 50–94% in WHO Grade 3 tumors, underscoring the aggressive nature of higher-grade disease [8]. Collectively, these retrospective analyses support an important prognostic role for tumor biological and clinicopathologic factors. WHO Grade 3 histology, increased proliferative activity as reflected by elevated Ki-67/MIB-1 labeling indices, invasion into the brain parenchyma or bone, subtotal resection, presentation with recurrent disease, and larger tumor size have consistently been identified as key predictors of recurrence [9,10,22]. In a cohort of patients treated with postoperative radiotherapy that included 66 WHO Grade 2 and 10 WHO Grade 3 meningiomas, Kim et al. reported that the crude rates of progression and/or recurrence differed markedly according to pathological and biological factors [9]. Specifically, progression or recurrence occurred in 80% of patients with Grade 3 histology, in 40% of those with brain and/or bone invasion regardless of WHO grade, and in 20% of patients with a proliferative index ≥ 3%, also independent of WHO grade [9]. Similarly, Kent et al. found that a Ki-67 index exceeding 10% was associated with a trend toward poorer progression-free survival among patients with atypical or malignant meningiomas receiving adjuvant radiotherapy [21]. In addition, Hoffmann et al. evaluated 31 patients treated with adjuvant (n = 11) or salvage radiotherapy (n = 20) and demonstrated that tumors with MIB-1 expression above the median value of 8% showed a greater likelihood of subsequent tumor regrowth compared with those with lower MIB-1 expression [23]. In contrast, our descriptive analysis did not demonstrate a clear relationship between the Ki-67 proliferation index and recurrence. Notably, three of the four patients without recurrence also had Ki-67 indices ≥10%, further indicating that an elevated Ki-67 index alone did not clearly discriminate between recurrent and non-recurrent cases in this small cohort. The median Ki-67 index was identical in patients with and without recurrence, and recurrence did not show a consistent increase across these Ki-67 categories. Given the very small sample size, these findings should be interpreted cautiously and do not exclude a potential prognostic role of Ki-67.

Importantly, prior studies have demonstrated that the majority of tumor recurrences arise within high-dose irradiated regions rather than at treatment margins, raising the possibility that biological factors may contribute to in-field treatment failure [23,24]. The recurrence patterns observed in our cohort are consistent with these previous observations. In the present study, most treatment failures occurred unequivocally within the high-dose region, with only a single patient experiencing both marginal and out-of-field progression. These findings raise the possibility that tumor biological factors may have contributed to the observed in-field recurrences, although their relative contribution cannot be determined from this small cohort. Other factors, including microscopic tumor extension beyond the delineated target, limitations in target delineation and postoperative imaging, and biological heterogeneity, may also have influenced the observed recurrence patterns. Our patient population was enriched with adverse prognostic features, including a high proportion of WHO Grade 3 tumors and multiple cases of recurrent disease at presentation. Although several patients had elevated Ki-67 indices, our descriptive analysis did not demonstrate a clear association between Ki-67 and recurrence in this small cohort. These factors have been associated with early treatment failure and may partly explain the comparatively low local control rate observed in this series. Previous reports have suggested that a Ki-67 index exceeding 10% is associated with an increased risk of recurrence even after gross total resection in WHO Grade 2 meningiomas [22]. In our cohort, one patient with WHO Grade 2 disease who underwent gross total resection and had a Ki-67 index of 31% developed an in-field recurrence; however, this individual case should not be interpreted as evidence of an association between Ki-67 and recurrence. Given that similar cases have been described in the literature, such findings support further investigation of proliferative indices as part of recurrence-risk assessment, even in ostensibly low-risk surgical scenarios. Patient 1 warrants particular consideration in interpreting the local control outcomes. This patient had WHO Grade 3 disease, the largest tumor volume in the cohort (103.7 cm^3^), and the highest Ki-67 index (69%), representing a particularly unfavorable prognostic profile. In addition, because of prior prophylactic whole-brain irradiation, the prescribed dose was limited to 45 Gy in 25 fractions, which was lower than that administered to the other patients. Therefore, the in-field recurrence observed in this patient may have been influenced by both adverse tumor characteristics and the lower deliverable radiation dose. Given the small sample size, this individual case represents an important potential confounder when interpreting the relatively low local control observed in the present series.

In this context, it is important to interpret individual recurrence patterns within the framework of contemporary margin selection principles. Contemporary consensus documents (e.g., ANOCEF and ESTRO-ACROP, NCCN) emphasize anatomically constrained, disease-specific margins rather than large circumferential expansions. Reported recommendations vary by guideline and clinical scenario, with postoperative CTV expansions commonly in the 5–20 mm range for WHO grade 2 and 3 meningiomas depending on tumor location and extent, always respecting natural anatomic barriers [7,25,26]. In our cohort, CTV margins of 5–10 mm were used, and only one marginal recurrence was observed. This case involved intentional restriction of the irradiated volume due to normal brain tolerance; therefore, in selected cases of WHO Grade 3 disease with suspected microscopic extension, careful consideration of margin selection may be warranted.

The predominance of in-field recurrence raises the important question of whether dose escalation or treatment intensification may benefit selected patients. Among historical dose–response studies using photon and mixed photon–proton approaches, the report of Hug et al. was noteworthy. They reported improved local control at target doses of ≥60 Gy for atypical and malignant meningiomas, as well as improved survival at doses of ≥60 CGE (cobalt gray-equivalent) in malignant meningiomas [27]. In their series, mixed photon–proton protocols enabled dose escalation up to 68–72 Gy/CGE; however, symptomatic radiation-related toxicity was observed in a subset of patients, highlighting the need for careful patient selection and dose optimization when pursuing treatment intensification. Similarly, Boskos et al. reported that in patients treated with combined photon and proton radiotherapy, a total dose exceeding 65 Gy was associated with a trend toward improved overall survival on univariate analysis [28]. More recently, Zeng et al. reported improved local control and progression-free survival with dose-escalated radiotherapy (≥66 Gy equivalent dose in 2-Gy fractions using an α/β = 10) delivered using modern techniques, including IMRT-based simultaneous integrated boost and, in selected cases, sequential stereotactic boost with Gamma Knife Icon [29]. Particle therapy, including carbon-ion radiotherapy, may also represent a future option for selected high-risk meningiomas; however, its role remains investigational [30]. Although data remain limited, these techniques illustrate potential strategies for improving local treatment efficacy in selected high-risk meningiomas. In particular, the potential role of dose intensification in patients with high-risk features, such as Grade 3 disease, subtotal resection, or markedly elevated Ki-67/MIB-1 indices, warrants further investigation in prospective studies.

In addition to technical refinements in radiotherapy, several emerging approaches may help address the risk of recurrence in biologically aggressive tumors. Novel modalities such as boron neutron capture therapy (BNCT) have been explored in heavily pretreated cases [31,32]. Moreover, molecular risk stratification is increasingly relevant; DNA methylation profiling provides robust prognostic information beyond histology, and malignant methylation classes have been associated with early recurrence in heterogeneous cohorts, independent of histological grade and treatment modality [33]. By contrast, systemic therapy remains an unmet need, with limited durable responses reported to date [34]. Collectively, these developments highlight the potential value of investigating treatment strategies that integrate biological risk profiling with individualized local and multimodal approaches.

Although conventional postoperative radiotherapy remains an established treatment approach, the present study cannot determine which patient subgroups may benefit from alternative radiation doses or treatment strategies. In patients with higher-risk features, such as Grade 3 histology, high Ki-67 indices, invasion into the brain parenchyma or bone, prior recurrence, or subtotal resection, the potential role of individualized treatment approaches—including dose escalation, stereotactic boosts, or advanced particle therapy, potentially guided by molecular risk stratification—warrants further investigation in larger, appropriately designed studies.

### Limitations

This study has several limitations. First, the retrospective design and small cohort size substantially limit the statistical robustness of the analysis and restrict the generalizability of the findings. Consistent with this limitation, the 95% confidence intervals around the Kaplan–Meier estimates were wide; for example, the 3-year LC and OS estimates had 95% CIs of 7–71% and 38–94%, respectively, underscoring the substantial uncertainty associated with these estimates. In addition, the present cohort included a substantial proportion of patients with high-risk disease characteristics, including WHO Grade 3 histology, recurrent disease, high Ki-67 indices, and invasive features. Although all consecutive patients meeting the predefined eligibility criteria were included and no additional exclusion criteria were applied, the resulting case mix was enriched for aggressive disease features. This may have contributed to the relatively unfavorable local control observed in our series and limits the generalizability of the findings to the broader population of patients with WHO Grade 2–3 meningiomas. Accordingly, the present results should be interpreted in the context of this high-risk cohort. Second, as this single-institution study spanned an extended time period, heterogeneity in surgical practices, imaging protocols, and radiotherapy delivery was unavoidable. Radiotherapy techniques evolved during the study period, with patients treated using three-dimensional conformal radiotherapy (3DCRT) and intensity-modulated radiotherapy (IMRT), including volumetric modulated arc therapy and helical TomoTherapy. Although the basic principles of target delineation, including the CTV margin, remained generally consistent, differences in treatment planning systems and delivery techniques may have affected dose conformity and target coverage and consequently may have influenced local control and the observed patterns of failure. Because of the small number of patients, the potential effect of radiotherapy technique on treatment outcomes could not be evaluated separately. Third, local failure was assessed based on serial radiographic changes without predefined quantitative or volumetric criteria, such as RECIST or RANO, which may have introduced some subjectivity in the assessment of progression. Fourth, DNA methylation classification was not performed in this cohort. Therefore, although TERT promoter mutation and CDKN2A/B homozygous deletion were assessed, comprehensive molecular classification could not be incorporated into the present analysis. Fifth, the follow-up duration was heterogeneous, and several patients had relatively short observation periods, raising the possibility of underestimating late recurrences, which are not uncommon in high-grade meningiomas. Despite these limitations, our findings highlight clinically relevant challenges in the postoperative management of high-grade meningiomas and underscore the need for well-designed, prospective, multi-institutional studies incorporating standardized imaging, advanced radiotherapy techniques, and molecular profiling to refine treatment strategies and improve outcomes.

## 5. Conclusions

In this small, single-institution exploratory series, local control after postoperative radiotherapy was limited, with most recurrences occurring within the high-dose region. These findings raise the possibility that tumor biological factors may contribute to treatment failure despite postoperative radiotherapy; however, the small sample size, retrospective design, treatment heterogeneity, and absence of comprehensive molecular risk stratification preclude definitive conclusions. These observations should therefore be regarded as hypothesis-generating and require validation in larger multicenter cohorts incorporating contemporary molecular risk stratification and standardized treatment approaches.

## Figures and Tables

**Table 1:** Clinical Characteristics and Outcomes after Postoperative Conventionally Fractionated Radiotherapy for Grade 2–3 Meningiomas.

No.	Age	Sex	Location	Presentation	Volume (cm^3^)	Resection (Simpson Grade)	Grade	Ki-67 Index (%)	Brain Invasion	Bone Invasion	RT Dose (Gy/fr)	Recurrence
1	38	M	Convexity	Primary	103.7	GTR (I)	3	69	−	+	45/25	Local recurrence (In-field)
2	66	F	Parasagittal	Recurrent	37.1	GTR (I)	3	40	+	+	60/30	Local recurrence (Marginal + Out of field)
3	75	M	Skull base	Recurrent	48.4	GTR (II)	3	14	+	−	60/30	Local recurrence (In-field)
4	58	F	Suprasellar	Primary	52.8	STR (IV)	2	5	−	−	60/30	Local recurrence (In-field)
5	75	M	Parasagittal	Recurrent	28.1	GTR (III)	2	7	+	−	60/30	Local recurrence (In-field)
6	44	M	Lateral ventricle	Primary	54.8	GTR (I)	2	31	+	−	60/30	Local recurrence (In-field)
7	85	F	Parasagittal	Primary	56.9	GTR (III)	3	30	+	−	60/30	−
8	80	F	Frontal lobe	Primary	41.6	GTR (I)	2	30	−	−	60/30	−
9	82	M	Frontal lobe	Primary	44.7	GTR (II)	2	15	+	−	60/30	−
10	76	M	Parasagittal	Recurrent	57.1	GTR (III)	2	5	+	−	60/30	−

Abbreviations: M, male; F, female; GTR, gross total resection; STR, subtotal resection; RT, radiotherapy; Gy, Gray; fr, fraction(s); +, positive; −, negative.

**Table 2:** Dosimetric characteristics of recurrent lesions.

Patient No.	Recurrence Pattern	Recurrent Tumor Volume (cm^3^)	V90 (%)	D95 (Gy)
1	In-field	8.12	100.0	44.2
2a	Marginal	7.22	1.5	7.1
2b	Out of field	2.15	0	2.1
3	In-field	16.74	95.1	54.5
4	In-field	48.73	87.5	48.8
5	In-field	4.99	100	60.7
6	In-field	3.39	100	60.5

Abbreviations: V90, percentage of the recurrent tumor volume receiving at least 90% of the prescribed dose; D95, dose received by at least 95% of the recurrent tumor volume. In Patient 2, the marginal (2a) and out-of-field (2b) recurrent lesions were analyzed separately.

**Table 3:** Summary of Selected Clinical Studies of Postoperative Conventionally Fractionated Radiotherapy for World Health Organization (WHO) Grade 2–3 Meningiomas.

Author/Year	Study Type	n	Grade	Extent of Resection	RT Technique	Median Total Dose (Gy)	Overall Survival (%)	Local Control (%)
RTOG 0539, 2020 [18]	Phase II	53	G2: 36% G3: 41% (Image-only recurrence: 23%)	Subtotal: 30% Gross total: 47% (Image-only recurrence: 23%)	IMRT	60	78.6 (3-yr)	68.9 (3-yr)
EORTC 22042-26042, 2018 [19]	Phase II	56	G2: 100%	Gross total: 100%	3DCRT/ IMRT	60	98.2 (3-yr)	NA
Detti B, 2013 [20]	Retrospective	68	G2: 75% G3: 25%	Subtotal: 20% Gross total: 80%	2D/3DCRT	54.6	74.1 (5-yr)	NA
Present Study	Retrospective	10	G2: 60% G3: 40%	Subtotal: 10% Gross total: 90%	3DCRT/ IMRT	60	79 (3-yr)	39 (3-yr)

Abbreviations: RT, radiotherapy; G2, Grade 2; G3, Grade 3; 2D, two-dimensional; 3DCRT, three-dimensional conformal radiotherapy; IMRT, intensity-modulated radiotherapy; n, number of patients; yr, year(s); NA, not available or not reported. Comparisons across studies are descriptive only. No formal statistical testing was performed because of substantial heterogeneity in patient selection, extent of resection, radiotherapy dose and fractionation, and reported endpoints (e.g., PFS, local control, and DFS). In EORTC 22042-26042, conventionally fractionated external beam radiotherapy was predominantly used, although stereotactic fractionated radiotherapy was permitted in selected cases.

## Data Availability

The data are not publicly available due to privacy and ethical restrictions. The study protocol approved by the Institutional Review Board does not permit the sharing of individual-level patient data with third parties.
